# Exploring the current state of technology transfer in the United States: perspectives and improvement strategies from the experts

**DOI:** 10.3389/frma.2024.1376185

**Published:** 2024-05-20

**Authors:** Jordan Eidlisz, Isabelle von Simson, Gabrielle Gold-von Simson

**Affiliations:** ^1^Department of Medicine, New York University Grossman School of Medicine, New York, NY, United States; ^2^Department of Medicine, State University of New York Downstate College of Medicine, Brooklyn, NY, United States; ^3^Department of Liberal Arts, Tulane University, New Orleans, LA, United States

**Keywords:** technology transfer, commercialization, biomedical devices, biotechnology, entrepreneurship

## Abstract

Technology transfer (TT) is a necessary, yet complex process to convey and disseminate scientific knowledge to the commercial sector. However, multiple barriers in TT can impede commercialization and innovative progress. To cultivate a deeper understanding, we conducted five interviews with strategic, elite leaders in different areas of TT in the United States. Experts shared their perspectives on the current state of TT, what needs improvement, and potential solutions to enhance the TT landscape, with a focus on biotechnology and medical devices. The formation of strong management teams, a comprehension of the regulatory, reimbursement, and funding pathways and policies, and thorough market assessments were noted as key aspects for venture success. Collaboration with Technology Transfer Offices (TTOs), industry experts, and strategic partners are also essential to support academic innovators and guide them throughout the complex commercialization process. There is agreement that a venture should have a defined vision and clear goals with a robust business case for the innovation; early involvement of TTOs is essential. Comprehension of the complexities and key facets of TT, while also streamlining the process, will better position biomedical innovators for success.

## Introduction

**Figure 1:**
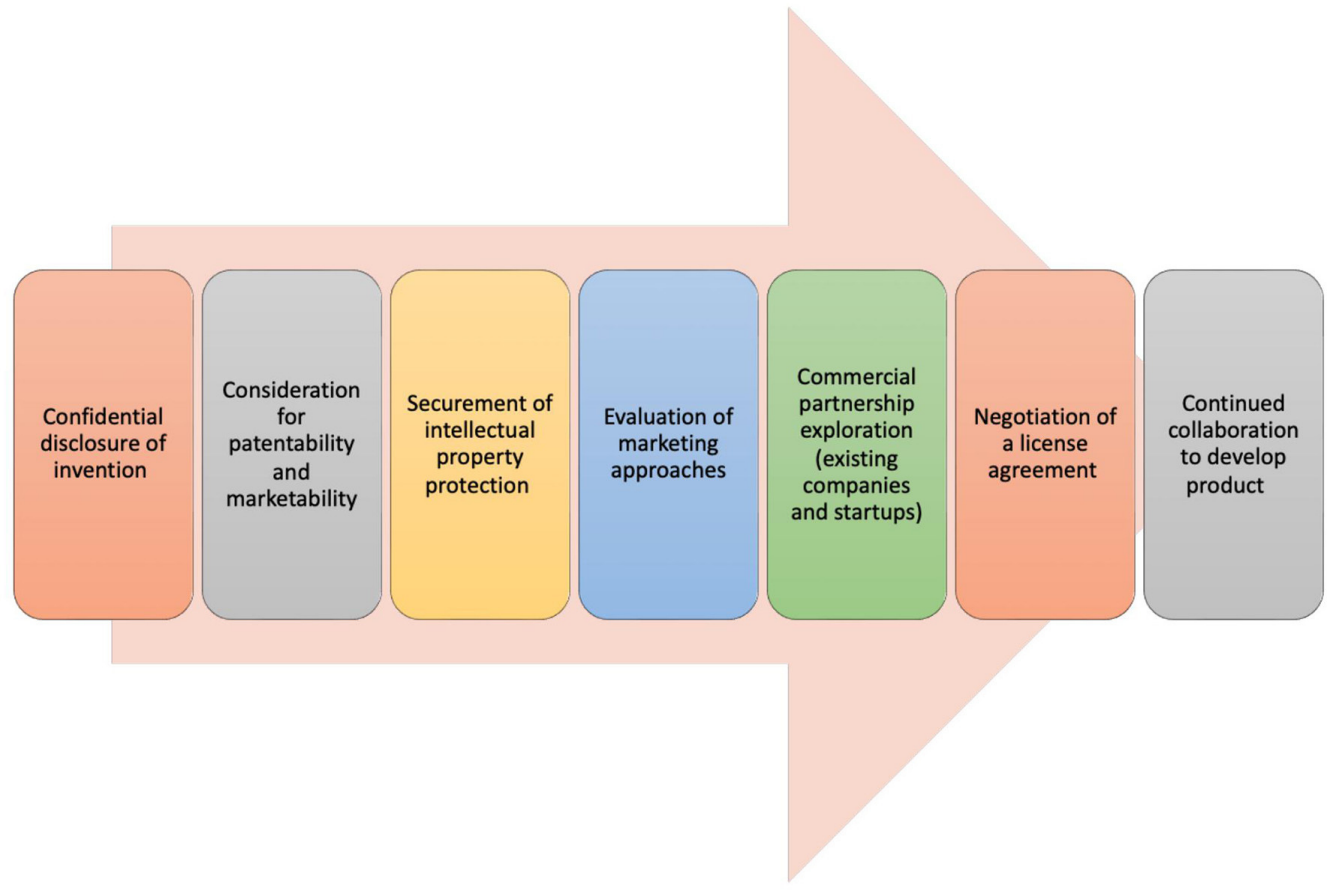
Technology transfer process. The process of technology transfer involves a sequence of stages, each necessitating close cooperation among a technology transfer office, the inventor, and various external entities and experts (Centers for Disease Control and Prevention, 2023; New York University, 2023).

Technology transfer (TT) describes a process of transferring scientific knowledge, innovations, and technologies developed within academic, research, or government institutions to the commercial or industry sector. This transfer aims to facilitate the practical application and commercialization of scientific discoveries for broader public benefit, which in turn fosters further innovation and economic development. The TT process involves the protection and licensing of IP, forming partnerships, and/or establishing companies (Figure 1). To bridge the gap between scientific research and real-world applications, many research organizations and universities in the US have established Technology Transfer Offices (TTOs). With a significant amount of federal funding channeled into academic research at universities, and the potential subsequent licensing revenue generated, TTOs are essential components of university systems. They have contributed to an increased number of startups launched from universities and fostered more partnerships between universities and industry, which includes many within the biotechnology and biomedical device realms (Siontorou and Batzias, 2010; Pestonjamasp, 2012; Hait and Stoffels, 2021). However, despite advancements and process improvements, there are still numerous challenges in TT (Milken Institute, 2017; Ip Watchdog, 2020; A Conversation on Technology Transfer, 2022).

## Challenges and solutions

Most biomedical/life sciences ventures fail, not necessarily due to a lack of ingenuity or potential, but because of a myriad of obstacles that impede successful commercialization, and a lack of guidance required to successfully navigate these obstacles. As many as 90% of biotechnology startups fail (Chakraborty et al., 2023). One of the most significant barriers is the perceived lack of support from academic institutions, which are often the birthplace of innovative healthcare technologies within the US (Commercialisation and Entrepreneurship - an Academic Viewpoint, 2022; DeSantola, 2022). Improvements in TT interfaces can streamline the process, making it easier for biomedical innovators to navigate the path to market. Education and training for early-stage researchers and founders equips them with the knowledge and skills needed to overcome hurdles in the complex biotechnology and medical device commercialization process and has been shown to improve competency in entrepreneurship and commercialization (Vizgan et al., 2022, 2023; Eidlisz et al., 2024). Additionally, increased support from academic institutions and other partnerships can provide the resources and backing necessary for success (Siontorou and Batzias, 2010; Hait and Stoffels, 2021).

## Interview methodology

We sought to conduct qualitative structured elite interviews with US strategic leaders in TT to identify and address the challenges biomedical innovators face. Interviewees included directors of TTOs at top universities (public and private) within the US, a biotechnology consultant, and a biotechnology intellectual property (IP) attorney, all expert representatives of the groups that innovators interact with on the pathway toward product commercialization. These interviewees were specifically selected since they are part of a well-informed elite spanning early-stage discovery to IP in commercialization. As difficult to access key informants, they provide a unique expert perspective on the TT process. Questions for the interviewees were developed with biomedical entrepreneurship, drug development, evaluation science, and clinical research content experts. Interviewees were asked to participate via email and were interviewed over email and/or via Zoom averaging 30–45 min in length. Responses were analyzed and aggregated by theme (biotechnology and medical device development essentials, common reasons for venture failure, comparisons between different commercialization advisory groups, improvements for future TT, and policy impact on innovation and development). Many interviewers gave similar responses, which are written out in aggregate. However, individual interviewees are quoted in the interview write up for specific responses they provided or topics they emphasized. Our goal was to gain perspective on the current state of TT for life sciences (medical device, biotechnology, drug development) within the US and discuss what works, what needs improvement, and how we can bridge the gap between academia and industry to foster successful TT. We also explore potential solutions that can be implemented to enhance the TT landscape, focusing on medical devices and their associated challenges.

### Interviewees

- **Interviewee 1 (I1):** Director of Life Sciences Technology Transfer at NYU Grossman School of Medicine/NYU Langone Health.
- **Interviewee 2 (I2):** Executive Vice President of Reimbursement, Value Generation and Market Access at Pria Healthcare.
- **Interviewee 3 (I3):** Chief IP Counsel at Aldevron.
- **Interviewee 4 (I4):** Associate Vice President of Tech Launch Arizona at the University of Arizona.
- **Interviewee 5 (I5):** Senior Associate Provost, Chief Technology Development Officer at Harvard University.

### Interview questions

1. What are aspects of a medical device/other venture that make you think it's a good candidate for commercialization?
2. What advice would you give to founders or researchers who want to commercialize their medical devices?
3. What are the top reasons you've seen that have caused ventures to fail?
4. If a researcher isn't interested in creating a company themselves, but would like their idea to be commercialized, is there a way for them to join with an industry partner?
5. What are some unique challenges faced by researchers looking to commercialize biomedical device products?
6. What are some differences you find between private consultants, biotech consultants, and university TTOs?
7. What would you say is the best way to interface with other institutes/offices within the university, with other universities, as well as with external consultants?
8. What do you think is lacking (if anything) in guidance from TTOs, specifically in regards to medical devices? What solutions would you recommend to address these issues?
9. When would you say it's better to use a private consultant as opposed to a university TTO? Is there a way to leverage both and reduce overall costs?
10. How can policies influence or direct founders?

## Responses by theme

### Biotechnology and medical device development essentials

A strong management team should be assembled that has experience in the commercialization of novel medical technology. This team must clearly understand the strategic intent of the technology and what types of funding will be needed. They must go above and beyond; “ventures should not simply stop at what the FDA requires.” (I2) Conversations should be held with the inventor(s) to determine how their goals relate to the product.

“Not every idea should or needs to be patented.” (I4) The product itself should meet an unmet medical need, provide value to users, and resolve a particular issue for the target population. “The innovation should have a novel approach, or a new mechanism of action, that's backed by vigorous lab research.” (I5) Research must be conducted into the patent landscape that surrounds the invention to determine if there's a path for viable IP protection. Simultaneously, market research should be done to determine potential license targets, competitors, and whether there is a patient population that actually needs the product.

A commercialization pathway should then be chosen, and one must decide whether to license to an existing company or form a startup. “Innovators must consider the personal time commitment required and assess how their ongoing research and career will be affected.” (I4) Successfully licensing their innovation to an existing company substantially lowers their time commitment but still leads to monetary return, personal gratification, societal impact, and often faster market access. “Inventors should reach out to colleagues of theirs who have successfully commercialized their own innovations to obtain advice.” (I5)

The realm of regulatory approval is incredibly difficult to navigate, making partnerships a crucial element of the commercialization process. Innovators should reach out to a TTO, potential investors, and consultants to obtain guidance and build a strong and experienced team. Moreover, a venture should have a defined vision, clear goals, and a robust business case for innovation, patent viability, and IP protection. All members of the management team should consider commercial, regulatory, marketing, and reimbursement strategies early on in product development. In-depth market assessments should be conducted that focus on a specific patient population, and the market potential should be evaluated with an assessment of competitors in the field. In addition, a target product profile and value proposition should be created. “TTO's with venture development resources often lead or support many of these activities with or for the innovator.” (I1) Researchers can partner with existing companies if they are interested in commercializing their ideas through licensing agreements or asset acquisition. “Startups and inventors can attract larger industry partners through the securement of IP, participation in trade shows, and positive research outcomes.” (I3) “Strategic companies also actively seek external innovations for growth, and often invest in, and acquire, startups.” (I2)

“Typically, TTOs lead the patent process, conduct market analysis, and identify and facilitate connections with companies for potential licensure.” (I1) The TTO will also actively negotiate deals on behalf of the inventor and their academic organization. “The inventor is an integral part of this process, as they serve as an expert on the technology and help set the goals for commercialization.” (I1) Although more uncommon, startups can be formed without the direct involvement of the inventor. This is a difficult path and requires finding the right team with the required devotion to the potential of the IP. However, this often still requires support and input from the inventor.

The time-consuming and expensive FDA approval process is a key distinction between medical devices, drugs, and other technology products. Medical devices derived from biotechnology face their own unique regulatory pathways and processes, such as device specific issues that include software as a service and engineering solutions. Depending on their classification, medical devices may require FDA clearance via the *de novo* process, 510(k) approval, or if a designated class III device, must provide robust clinical data for FDA approval against existing standards of care. “A prototype is crucial to demonstrate medical device functionality.” (I3) This requires a suitable shop, funds, and/or university resources. “Medical devices have limited venture capital support due to their lower profit potential compared to pharmaceuticals.” (I5) This leads to not only less funding, but a less organized and linear funding process for medical devices compared to the funding pathways for drugs. In addition, medical devices and drugs have distinct reimbursement mechanisms, pricing strategies, coding methods, coverage, and payment structures. “Different stakeholders are involved in managing benefits for drugs (pharmacy benefit managers) and medical devices (medical policy committees and various departments).” (I2)

### Common reasons for venture failure

Leadership challenges, such as inadequate or inexperienced leadership, a poorly constructed team, a lack of clear direction, and disagreements among leadership, can derail a venture. Financial hurdles, such as a lack of access to capital, insufficient funding, premature depletion of funds, or failure to choose the right investors, can also stop development. “A common cause of failure is the inability to secure regulatory approval, keeping the product off the market.” (I3) Even if a product makes it to market, if the idea was insufficiently vetted with inadequate consideration of risks, or unvalidated assumptions were made, such as pricing the product too high with minimal data or a lack of product market fit, the product can fail. “Another common pitfall is a poor understanding of the reimbursement pathway or a lack of reimbursement strategy.” (I2) This leads to unclear payment strategies for stakeholders and providers and can lead to the depletion of resources due to delayed reimbursement.

### Comparisons between different commercialization advisory groups

TTOs operate within many US universities to commercialize government-funded research innovations created by their faculty. “They aim to benefit the public by turning bench research innovations into commercializable products.” (I4) “The revenue obtained from university driven products goes back into research, development and to inventors themselves.” (I1) TTOs also collaborate with external partners to transfer technology and receive additional guidance in specific areas. In the US, academic institutions generally own IPs developed using federal funds, which makes TTOs the primary route for moving forward with commercialization.

Multiple models and programs are used by TTOs to facilitate connection and collaboration within university departments, many of which are specific to their representative university. “Some examples include lunch-and-learns to engage faculty and staff on commercialization and research topics, and educational programs/courses that raise awareness of TTOs, university resources, and commercialization processes.” (I4)

When interacting with other universities, organizations such as the Association of University Technology Managers (AUTM) play a pivotal role in the distribution of best practices and setting standards. “Scientists are intrinsically collaborative, and universities often establish inter-institutional agreements for handling IP that arises from joint inventions.” (I1) Scientists from various universities will often collaborate freely and provide resources, templates, and insights to one another. Consultants, experienced business professionals, and entrepreneurs are often recruited by TTOs within universities to give lectures/seminars and share their valuable knowledge on commercialization processes.

Private consultants seek out novel innovations and technologies and work independently or for consulting firms. “They focus on the transfer of technologies between parties, and typically cover a broad range of technology types, industries, and disease areas. Biotechnology consultants focus on technologies related to biotechnology and life sciences, and specialize in a few specific disease areas or product types which they become experts in. Private consultants and biotechnology consultants have a fiduciary responsibility to their company and/or shareholders to earn profit.” (I2)

Private consultants increase costs but can be crucial for the development and long-term success of medical technology because of their expertise. In this sense, private consultants are often brought in when the TTO lacks expertise in a specific area or if a technology is crucial to ongoing work and all TTO avenues have been explored. “Selectively using external consultants on limited projects with specific goals (e.g., research an FDA approval pathway) can benefit the TTO (easier to license) and the startup (knowledge of how to proceed).” (I4) Oftentimes, the decision whether to use private consultants depends on the university's budget. Properly guiding innovators is an iterative, multi-year endeavor (5–9 years) that may require external expertise at several points throughout. Once a technology has been deemed feasible in terms of reimbursement, marketability, patient population, and other commercialization considerations, it may be time to hire consultants or industry experts to help further guide the process.

### Improvements for future TT

“Institutions should create more offices with dedicated development funds focused on medical devices to provide inventors with the necessary financial support.” (I4) Increased TTO expertise and support on the FDA approval process can shorten the commercialization timeline. “Realistic valuation methods for medical devices should be developed and utilized to ensure inventors receive fair compensation and attract investors.” (I3) “TTOs should be encouraged to collaborate with industry experts, clinical experts, external consultants, and epidemiologists, who can provide valuable guidance and mentorship to innovators.” (I2) Institutions also should offer specialized educational programs for clinician researchers on the commercialization process, a domain that traditional scientific degree programs lack. “As opposed to researchers who are dedicated to the lab, clinicians spend most of their time engaged in clinical activities and can only innovate on the side when their availability permits. Institutions must be aware of this and do whatever they can to help shepherd physician scientists' ideas through the commercialization process.” (I1)

### Policy impact on innovation and development

Policies at various levels play a significant role in guiding founders along the commercialization process. At the Federal level policies such as the Bayh-Dole Act encourage technology commercialization at universities. State level policies that promote benefits, such as tax breaks and incubators, further support scientific founders. Founders must understand and consider regulatory policies put in place, and the precedents set, by the FDA. “Additional commercialization policies, such as those related to reimbursement, need to be considered early on, and throughout, the design, development, and commercialization processes.” (I2) An example involves Transcutaneous Electrical Nerve Stimulation (TENS) devices. Certain startups have tried to avoid the classification of their product as a TENS device due to the unfavorable reimbursement pathway (Medicare, 2023).

“University policies that involve revenue sharing incentivize innovation and benefit founders, and awards for innovation and entrepreneurship can motivate faculty engagement.” (I1) “A culture of innovation and support for faculty and staff within a university can drive activity and engagement in commercialization efforts.” (I4) Conflict of interest policies are essential to guarantee that product research isn't influenced, or has the appearance of being influenced, by the startup's objectives. Some university policies impose restrictions on founders, such as not allowing them to serve on the board of directors or management teams while they remain full-time researchers. Founder engagement relies on clear rules and effective management.

## Discussion

Several critical aspects come into play when one considers a medical device or other venture for commercialization. It's essential to conduct comprehensive research into the patent landscape, assess market viability, understand potential competition, and secure strong IP protection. A strong and experienced management team that understands the complexities of bringing novel medical technologies to market is critical to venture success. Leaders must be able to recognize and anticipate funding requirements and navigate regulatory demands, especially from the FDA.

Some common causes of venture failure include unfounded assumptions about pricing, inexperienced leadership, insufficient funding or capital, a lack of knowledge of regulatory and reimbursement pathways, and the lack of a coherent reimbursement strategy. Conversely, comprehensive market assessments, an understanding of target patient populations, and crafting a value proposition tailored to specific clinical outcomes are crucial steps associated with venture success. Innovators and partners must consider all these factors in the early stages of their venture.

In terms of funding and strategic partnerships, larger pharmaceutical companies often seek external innovations to integrate into their portfolios and incubate. Venture funding is sought from investors and strategic partners, who offer not only capital, but mentorship and guidance to startups.

The commercialization pathways for medical devices and biotechnology have various challenges. Medical devices face distinct reimbursement pathways and market access processes compared to biotechnologies, particularly in terms of pricing, coding mechanisms, and coverage strategies. Biotechnology products typically undergo placebo-based randomized control trials, while medical devices are generally compared to existing standards of care.

Individuals who work as biotechnology consultants, private consultants, and within university TTOs face unique experiences. Biotechnology consultants gain expertise on specific products, while private consultants handle broader disease areas and technology types. TTOs play a crucial role in the assistance of academic innovators and help guide an innovation from the bench to the market. Collaboration, especially with regulatory and clinical experts, accelerates successful commercialization. Although private consultants often cost more compared to TTOs, their expertise in specific fields can help expedite the commercialization pathway. All experts within these organizations provide insights into market access, reimbursement, and other essential areas in the commercialization process. Industry experts, including clinical and regulatory professionals, play a vital role in educating innovators and guiding them through the complex commercialization journey. Furthermore, educational programs offered by TTOs provide segmented consultation and insights into considerations for commercialization. Further incorporation of entrepreneurial education into biomedical curricula could enhance the effectiveness of tech transfer programs, as has been shown with the effectiveness of prior programs devoted to biomedical entrepreneurial education (Vizgan et al., 2022, 2023; Eidlisz et al., 2024).

The commercialization journey takes several years and requires meticulous planning and iterative adjustments. A practical example involving TENS devices illustrates the importance of various policies early in venture creation. Proactively addressing such considerations can prevent policy-related roadblocks. Other countries and worldwide regions have regulatory hurdles, commercialization policies, and challenges specific to their governance; we did not seek to explore this here. Expert interviewees for this article were all located at companies and universities based within the US. Future interviews with foreign TT experts can provide more comprehensive descriptions of TT on a global scale which is important for integration of data, collaboration beyond US borders, and solutions that can improve the TT process.

Despite their different roles in the TT domain, interviewees' perspectives aligned on much of the core essentials required to successfully bring a biomedical product from bench to market, what needs improvement within TT, and potential solutions to enhance the innovative landscape in the life sciences. Successful commercialization requires foresight and collaboration, comprehensive research, understanding regulatory processes, as well as alignment with university, and government policies. The formation of strong management teams, understanding regulatory, reimbursement, and funding pathways and policies, and thorough market assessments, were noted by our elite experts as key aspects for venture success. Collaboration with TTOs, industry experts, and strategic partners, in addition to educational programs focused on the science of translation, are needed to further support academic innovators. Understanding, streamlining, and improving the TT process will bridge the gap between scientific research and useful application, fostering an innovative climate in the life sciences to enable successful commercialization.

## Data availability statement

The raw data supporting the conclusions of this article will be made available by the authors, without undue reservation.

## Ethics statement

Written informed consent was obtained from the individual(s) for the publication of any potentially identifiable images or data included in this article.

## Author contributions

JE: Writing – original draft, Writing – review & editing. IS: Writing – original draft, Writing – review & editing. GG-vS: Writing – original draft, Writing – review & editing.
